# Triple aim improvement for individuals, services and society in dementia care: The DementiaNet collaborative care approach

**DOI:** 10.1007/s00391-017-1196-4

**Published:** 2017-02-20

**Authors:** M. S. Nieuwboer, A. Richters, M. A. van der Marck

**Affiliations:** 1 Radboudumc Alzheimer Center, Department of Geriatric Medicine, Radboud university medical center, PO 9101 (hp 925), 6500 HB Nijmegen, The Netherlands; 2 Donders Institute for Brain Cognition and Behaviour, Department of Geriatric Medicine, Radboud university medical center, Nijmegen, The Netherlands; 3 Department of Geriatric Medicine, Radboud university medical center, PO 9101 (hp 925), 6500 HB Nijmegen, The Netherlands

**Keywords:** Multidisciplinary team, Dementia, Collaborative care, Interprofessional collaboration, Primary care, Multidisziplinäres Team, Demenz, Netzwerkbasierte Versorgung, Interprofessionelle Zusammenarbeit, Erstversorgung

## Abstract

**Background:**

A redesigning of primary care is required to meet dementia patients’ needs. In the Netherlands, current dementia care still falls short in areas including ad hoc collaboration, lack of feedback on quality to professionals involved, and insufficient implementation of established multidisciplinary guidelines.

**Objective:**

DementiaNet is a collaborative care approach, which aims to reduce the burden of the disease on individuals, healthcare services and society via network-based care that encourages collaboration, enhances knowledge and skills and stimulates quality improvement cycles.

**Material and methods:**

DementiaNet was developed to support primary care networks through implementation of five core processes: network-based care, clinical leadership, quality improvement cycles, interprofessional practice-based training and communication support tools, following a stepwise tailor-made approach. Alongside this, a mixed method study was designed to evaluate innovation and effectiveness.

**Results:**

Currently, 18 networks have been formed. These vary in quality of care and strength of collaboration due to local circumstances. Initial activities and goals of each network also vary, ranging from acquaintance to shared care plans. Ongoing research will identify barriers, facilitators and merits of the approach in increasing quality of care and ultimately improving outcomes for patient, carer, health service and society.

**Conclusion:**

Initial results show that clinical practice varies and the DementiaNet approach can lead to quality improvement. Complexity and variety of local care requires complex interventions and evaluation methods that account for this in order to safeguard the value for practice. Strict methodology lessens external validity.

## Zusammenfassung

**Hintergrund:**

Die Erfüllung der Bedürfnisse von Demenzpatienten erfordert eine Neugestaltung der Erstversorgung. In den Niederlanden bestehen noch Defizite bei der Demenzbetreuung wie etwa ad-hoc-basierte Zusammenarbeit, mangelndes Qualitätsfeedback gegenüber beteiligten Professionals und unvollständige Implementierung etablierter multidisziplinärer Richtlinien.

**Ziel:**

DementiaNet will die Belastungen durch eine Demenz für Individuen, Gesundheitsdienstleister und die Gesellschaft durch netzwerkbasierte Versorgung reduzieren, die Zusammenarbeit stimulieren, Wissen und Können erweitern und Qualitätsverbesserungen unterstützen.

**Material und Methoden:**

DementiaNet wurde entwickelt, um Erstversorgungsnetzwerke mit der Implementierung von 5 Kernprozessen zu unterstützen: netzwerkbasierte Versorgung, klinische Führung, Qualitätsverbesserungszyklen, interprofessionelles praxisbasiertes Training und Kommunikationshilfsmittel. Diese Netzwerke folgen einer schrittweisen, maßgeschneiderten Methode. Daneben wurde eine Mixed-Methods-Studie entworfen, um Innovation und Effektivität zu beurteilen.

**Ergebnisse:**

Gegenwärtig existieren 18 Netzwerke. Sie variieren aufgrund lokaler Umstände bezüglich Versorgungs- und Zusammenarbeitsqualität. Zuerst unterschieden sich Aktivitäten und Ziele jedes Netzwerks, vom Kennenlernen bis zu geteilten Versorgungsplänen. Die fortlaufende Forschung wird hindernde und fördernde Punkte unseres Ansatzes identifizieren und zeigen, wie die Versorgungsqualität und damit die Ergebnisse für Patienten, Versorgungsdienstleister, Gesundheitsdienste und die Gesellschaft gesteigert werden können.

**Diskussion:**

Erste Ergebnisse zeigen, dass die klinische Praxis variiert und der Ansatz von DementiaNet zu einer Qualitätsverbesserung führen kann. Die Komplexität und Vielfalt lokaler Versorgung erfordern diesbezüglich komplexe Interventionen und Evaluationsmethoden, um den praktischen Wert zu gewährleisten. Strenge Methodik vermindert die externe Gültigkeit.

## Brief introduction

The number of elderly people with cognitive problems who are still living at home is likely to increase. As a result, primary healthcare professionals will be increasingly required to manage and optimize treatment for dementia patients. This underlines the need to improve dementia care within primary care. We developed the DementiaNet collaborative care approach, which includes a gradual reorganization of care towards high-quality, network-based dementia care. The development, implementation, initial experiences and study design are described to evaluate the possible merits of this approach.

## Shortcomings of current dementia care

Although many initiatives have recently been designed, collaborative dementia care is still fragmented and far from optimal due to lack of disease-specific expertise and training and limited communication between healthcare professionals [1]. A collaborative approach could be especially important for dementia patients as manifestation of the disease is often complex and complicated by comorbidities, while loss of mental autonomy and disease awareness are specific for this disease, and determine specific care needs. Dementia patients have to cope, not only with dementia, but also with other chronic health and welfare problems. In a large Scottish study, 95% of all dementia patients also had relevant concurrent diseases [2]. Yet, collaboration between healthcare professionals is mainly scheduled ad hoc rather than structurally. This was also apparent in a Dutch study into the effectiveness of post-diagnosis dementia care of memory clinics versus general practitioners conducted in nine memory clinics [3]. In both study arms, the care process was relatively unstructured. Furthermore, care was insufficiently personalized and structured without formal assessment of individual problems and priorities or taking the individual context into account [4]. Personalization should also address informal carers, who are often faced with a high burden. Another limitation to current practice includes the lack of long-term monitoring of symptoms, signs, quality of life, caregiver burden, and feedback on quality of care and cost-effectiveness [5]. To tackle these shortcomings, the DementiaNet approach aims to reduce the burden of the disease for all involved in dementia care, including healthcare professionals, patients and their informal caregivers (quality of life, perseverance time), and societal (cost-effectiveness) impact.

## Development of DementiaNet

DementiaNet functions as an overarching umbrella that facilitates the organization, implementation and maintenance of primary care networks, which are in direct connection with secondary care facilities for dementia. It was designed to support these networks to become an independent, sustainable and interprofessional collaborative, in which members can provide better quality of care and achieve higher effectiveness. Primary care for dementia patients in the Netherlands is characterized by complex social and financial developments. Due to the high societal and economic impact of dementia, the Dutch Government, as many others, aims for high-quality and affordable dementia care. Between 2005 and 2016 changes were instigated through the financing of four successive national dementia and elderly care improvement programs. This created a nationwide regional network structure, deployment of dementia case managers and dissemination of multidisciplinary guidelines; however, incomplete implementation and lack of structural finance caused large variation in the acceptance and adherence to the new guidelines and regulations in clinical practice. Additionally, in 2015, the Dutch Government introduced radical reforms in the financial structure of primary healthcare, resulting in shifting responsibilities for welfare and care from national and regional levels to local governments at municipality level. Responsibility for welfare was transferred to local authorities. General practitioners (GP) act as gatekeepers for medical care and community nurses (CN) determine the amount of nursing care required. Case management is not yet structurally financed; therefore, funding varies between regions and case managers are not available for all dementia patients. This new financial arrangement has created much insecurity for healthcare professionals and institutes, as well as for patients and their carers in primary care practice. The DementiaNet approach was designed taking this healthcare complexity, shifting roles and variety in clinical practice into account. A stepwise, tailor-made and bottom-up approach was chosen. Various stakeholders were consulted in designing DementiaNet. Primary care professionals and representatives of elderly and dementia patients were interviewed on their experiences, barriers and facilitators in dementia care. The theoretical framework underlying DementiaNet includes collaborative network theories, such as the conceptual framework of partnership collaboration [6], which emphasizes the importance of addressing shared ambitions, mutual gains and relationship dynamics between network participants. We also applied best practice models on quality improvement, including the Improvement Model/Plan-Do-Check-Act (PDCA) [7] and Breakthrough Series Collaborative [8], and evidence from previously implemented collaboration models, e. g. the ParkinsonNet [9] and Healthy Aging Brain Care model [10, 11]. Finally, experiences from previous primary care network projects were used. For example, as the presence of active clinical leaders emerged as the key to successful implementation, clinical leadership was added as a central theme of DementiaNet [12].

## Central themes

The DementiaNet approach consists of the following five central themes. These core themes form the basis for all DementiaNet networks, as the starting point for a stepwise, tailor-made approach.

### Network-based care

Each DementiaNet represents a local interprofessional team that includes healthcare professionals from medical, care and social domains e. g. GPs, CNs, dementia case managers (CM), and welfare professionals (WP). A CM supports community-dwelling individuals with dementia and their caregivers during the care process, from the prediagnostic phase to nursing home admission. The CM regularly visits patients at home and coordinates medical and social care. The WPs support patients and carers with participation in the community. They also visit patients at home and organize activities in the community, such as day care activities. Together, these professionals form a network in a local neighborhood, which is characterized by the catchment area of the GP practice. Recent research findings about interprofessional collaboration in primary care [13] support the importance of a team vision, shared goals, formal quality processes, information systems and shared team spirit; therefore, development of collaboration and communication skills including all these aspects and jointly sharing responsibility for improvement of dementia care are key issues.

### Clinical leadership

In the primary care setting, organizational and personal barriers can hamper collaborative team efforts, for example, lack of trust, absence of shared goals and lack of opportunities to meet [14]. Strong clinical team leadership is important to facilitate low-level redesigning of work, and achieve quality and efficiency improvements [15]; therefore, in each local DementiaNet network, at least one network participant is recruited to lead connection and quality catalysis. This network leader or network connector, must be able to connect the different professionals and stimulate collaboration. As this is a new role for many professionals, we developed a leadership program to provide support to these primary care clinical professionals.

### Quality improvement cycles

DementiaNet network members are stimulated to use practical tools to enhance quality improvement of dementia care. The process of quality improvement begins with data acquisition to facilitate feedback reports on performance measurements [16]. An online questionnaire is distributed to the network participants. This questionnaire consists of multiple validated instruments, such as team skills, attitudes towards healthcare teams, prerequisites for collaboration [14] and knowledge about dementia. Furthermore, data on quality of care are gathered including a concise set of quality indicators derived from the Dutch multidisciplinary guidelines for dementia care [17, 18]. Benchmarking provides members with insights into their own quality compared to the average quality of care of all participating networks. The network is then encouraged to discuss quality feedback, select a problem for focus, formulate goals and design an action plan, according to the PDCA cycle [7]. This tailor-made approach stimulates a sense of urgency and ownership amongst network members towards improved care.

### Interprofessional practice-based training and learning

Based on the feedback on quality of local dementia care and the action plan, we support the organization of practice-based interdisciplinary training on topics selected by the network participants. In these training sessions, examples from daily clinical practice are taken, in which complex cases are discussed to ensure integration of knowledge and practice. Teamwork can also be the focus of training sessions, as team competency is important for collaboration, although frequently lacking as healthcare professionals are often not actively taught to cooperate.

### Communication

Successful collaboration in practice depends on clear and effective communication between the key disciplinary groups [19]; therefore, communication tools are provided. For example, an electronic communication tool for healthcare professionals and informal caregivers to discuss patient cases and coordinate actions. Additionally, an online community will enable interprofessional communication and networking between different local platforms, and secondarily, more specialized dementia expertise.

## Stepwise development of a DementiaNet network

DementiaNet networks are formed via a stepwise approach. The program for each network is tailored to the members’ own needs and priorities. This tailor-made approach requires the guidance of each DementiaNet team in applying the central themes. Various steps to support the network are undertaken over a 2-year period. As a wide variety of dementia care practice exists between regions, the DementiaNet approach must be adapted to local settings and needs. In some networks, team members already collaborate. Hence, these networks obviously require a different approach than those in which team members have never worked together before. In general, the following three steps are undertaken to form a network and enhance performance:

### Step 1: Recruitment of network leaders.

The DementiaNet team organizes training sessions comprised of interprofessional workshops that address the DementiaNet themes. DementiaNet is also promoted in various local, regional and national healthcare meetings and through printed and online publications [20] to encourage professionals to start a network.

### Step 2: Network leader forms local network.

If a potential network leader is interested to join the program, the network leader and DementiaNet coordinator assess the local situation together. Detailed insight into actual dementia healthcare provision in that specific community is crucial to optimize connection to other related healthcare initiatives. If the potential network leader can organize a group of interested professionals, preferably from medical, care and social services, the DementiaNet coordinator meets with this potential team to provide information about DementiaNet and gauge support. This step usually takes 3–6 months and requires the commitment of the potential network leader; it is a first test of the leadership of this individual’s competencies. So far 18 network leaders have succeeded in establishing a DementiaNet network, 10 are still in the process of organizing the network and 17 healthcare professionals were not able to engage other professionals to jointly start a network.

### Step 3: Implementation of the DementiaNet program.

This step encompasses the implementation of the central themes, according to an action plan with: monitoring of team performance, annual self-assessment of quality of care in the local network and interprofessional and practice-based education to enhance expertise.

Network leaders also join a leadership support program based on the UK National Health Service (NHS) healthcare leadership model [21]. This provides individual coaching and group session workshops to improve personal leadership skills. Regular meetings facilitate long-lasting collaboration and help develop a collaborative view on healthcare [14, 22] through open discussion of task coordination and responsibilities and conflicts of interests. Prerequisites for collaboration and reflections on team performance results are also discussed in local network meetings. During the 2‑year program all network members attend interprofessional training workshops, often twice a year. Network members select training topics themselves, for example on recognition of cognitive decline, dementia diagnosis, complex behavioral problems and shared decision making.

## Scientific evaluation

An evaluation study provides insight into the possible merits of DementiaNet. The longitudinal mixed methods multiple case study design is in line with evaluation methods used for complex interventions. All DementiaNet networks serve as a case in this study and are followed over time. Quantitative data are collected at baseline and annually and qualitative data are collected throughout the course of the study to gain in-depth knowledge on processes and experiences of people involved i. e. care professionals, patients and informal caregivers. The evaluation study commenced at the start of the first network in January 2015 and will be concluded in the second half of 2017.

From the concept of evidence-based healthcare [22] it follows that local resources should be invested in those programs that have been studied and found to be effective. Regarding novel health care delivery systems, this is of great importance, as innovations occur in complex environments with numerous stakeholders and external influences that make the effects difficult to predict. This high level of complexity also applies to DementiaNet, emphasizing the need for a mixed methods design, especially as the approach is tailored to each network. In addition, innovations such as DementiaNet, are impossible to evaluate before implementation [23], and so implementation and evaluation occur simultaneously. For this, data are gathered from multiple sources for each network. Firstly, each network is rated on their network-based maturity, based on yearly structured interviews with the network leader(s). The rating is performed based on a Dutch model, The Primary Care Maturity Model, in which the level of network-based functioning is rated as one of four levels on eight domains [24]. Secondly, online questionnaires are completed by network members on instruments, such as team skills and attitude towards dementia. Each network is also requested to complete a set of quality indicators of care, as described, including indicators related to diagnostics in primary care setting, involvement of case management, geriatric assessment, care plan, polypharmacy check, and emergency consultations. Lastly, paper-based questionnaires are send to informal caregivers of patients within the network, including instruments to measure quality of life [25, 26], caregiver burden [27–29], satisfaction with care [30], and health services utilization. In addition to these data sources, in-depth interviews with care professionals in the networks, as well as informal caregivers and patients are performed to gain more insight into experiences with the DementiaNet approach, identify other possible merits or challenges and to find opportunities to enhance the DementiaNet approach to fit each situation better. We use semi-structured interviews which are transcribed verbatim and subsequently coded independently by two trained researchers after which consensus is obtained to ultimately lead to overarching lessons. Quantitative and qualitative data sources will be combined to reflect on our hypothesis. We hypothesize that network maturity level will change differently for each network, depending on varying baseline situation and improvement actions. We expect that quality of care, as measured by the quality indicators, will be associated with the network maturity and will increase if the network maturity has increased. We also measure informal caregiver reported outcomes; however, we realize that the timeframe of the current evaluation study might be too short to indicate significant effect, especially as these outcomes are indirectly influenced by the organization of networks. From the data, trends are examined over time by means of growth models. Not only are measurements within each network investigated but data between different networks are compared to identify improvement patterns. This is facilitated by natural contrasts between networks, as each baseline level differs and will vary in development during the 2‑year course. Qualitative data enables us to explain findings and patterns. Additionally, specific elements of the approach are assessed for effectiveness, including the DementiaNet leadership program and communication between GPs and CNs, as key players within the networks.

## Initial experiences and results

The first generation of DementiaNet currently includes 18 networks, distributed throughout the Netherlands. These networks are comprised of an average of 10 care professionals, and range from 5 to 22. The most frequently represented disciplines are GPs, CNs, CMs, and practice nurses. Other disciplines include allied health care professionals, such as physiotherapists and occupational therapists, and welfare professionals. In five networks, volunteers, interested groups or carers of dementia patients participate as team members. In total, the healthcare professionals in these networks provided care for over 278 community-dwelling dementia patients at baseline. As expected, the networks varied considerably regarding their situation on enrolment. Some networks had already worked together intensively for a long time and had already established reasonable levels of collaboration and communication. Of the networks six worked together in a program for complex elderly patients before they entered the DementiaNet program. Contrary, the majority of health care professionals were still focused on getting to know each other and formulating agreements on sharing responsibilities in care processes. This variety between networks is also reflected in the quality indicators, which show a large heterogeneity and indicate that improvements are still needed in several domains.

In general, the PDCA method to design quality improvement cycles is appreciated by healthcare professionals, as it requires them to focus on one or two specific aims at one time, for which they can draw up a concrete action plan. Since these cycles are based on each networks’ own goals and priorities, a wide variety of improvement targets were defined, including: improvement of collaborative skills, increase knowledge on management of behavioral changes, implementation of shared care plans for all professionals involved, enhancement of diagnostic expertise in the general practice, and optimization of the format of multidisciplinary team meetings.

## Conclusion

With DementiaNet, we aim to work towards high-quality, network-based care. These networks are organized on a local level, including healthcare professionals from medical, care and social disciplines. Based on theory, literature and experiences, we designed a stepwise approach to increase the quality of dementia care, including multiple elements on quality improvement, interprofessional learning and collaboration, and clinical leadership. So far, our initial experiences and results confirm the effectiveness of this DementiaNet design, as a tailor-made integrated care innovation, directly built on the differences and needs in clinical dementia practice. Although, initially, we aim to enhance dementia care, the basics of DementiaNet are general and might also, therefore, serve as a model to increase quality of healthcare for other populations, for example, frail elderly and patients that require palliative care.

## Practical conclusions

More patients with dementia will live at home for longer periods of time, which highlights the need to improve dementia care within primary care. DementiaNet improves local collaboration amongst primary healthcare professionals to provide care for community-dwelling elderly with dementia and their informal careers. Our mission is to deliver added value for patients, caregivers, healthcare services and society, by realizing an innovative, cost-effective change in care processes, finely tuned for local, collaborating professionals. We engage patients and carers, and start from their perspectives, which we adopt in line with network and system-based methodologies. As many themes and activities are generally applicable, the DementiaNet approach might also serve as a model towards enhanced collaboration and quality improvement for other populations.
